# Understanding patient perspectives on breast cancer risk: a qualitative study to inform genetic testing communication in the primary care setting

**DOI:** 10.1007/s12687-026-00933-4

**Published:** 2026-08-13

**Authors:** Jennifer McGuire, Vanessa Diaz, Katherine Sterba, Kevin S. Hughes, Sarah Tucker Marrison

**Affiliations:** 1 College of Medicine, Medical University of South Carolina, Charleston, SC USA; 2 Department of Family Medicine, Medical University of South Carolina, 135 Cannon Street Suite 405 MSC 192, Charleston, SC 29425 USA; 3 Department of Public Health, Medical University of South Carolina, Charleston, SC USA; 4 Department of Surgery, Medical University of South Carolina, Charleston, SC USA

**Keywords:** Breast Cancer Risk, Genetic Testing, Patient Perspectives, Informed Decision-Making, Cancer Screening

## Abstract

Early breast cancer risk identification is essential for targeted screening and prevention. Provider uptake of risk-stratified screening remains low, often due to limited education on risk assessment, clinical time restrictions, and limited resources for systematic implementation. Patient perspectives on incorporating screening tools into routine care are not well understood. This study elicited patient experiences and preferences regarding risk assessment and genetic testing in primary care. Twenty semi-structured interviews were conducted with individuals at average and high breast cancer risk. The interview guide was developed using the informed decision-making framework. Participants reviewed a decision aid and provided feedback on risk and genetic testing communication. Interviews were transcribed and thematic analysis was conducted by two independent coders. Study participants had an average age of 44 with 45% of individuals self-reporting high-risk for breast cancer. Women across risk levels desired clear, empathetic communication with detailed screening and genetic testing information. Financial constraints, fear of results, and confidentiality were perceived barriers. Key benefits included informed decision-making and prevention potential. Participants emphasized accessible information, personalized communication, and clear follow-up plans. High-risk participants demonstrated more active information-seeking, desired ongoing support after testing, and emphasized family. Most found the communication guide and decision aid supportive for informed decision-making. Patient-centered communication and accessible, comprehensive information are key for communication of breast cancer risk. Addressing financial barriers and anxieties surrounding genetic testing will be critical for equitable uptake. Future work should consider these patient preferences in designing interventions to improve risk-aligned care.

## Introduction

The landscape of breast cancer prevention and early detection has evolved with increased importance of personalized risk assessment and genetic testing. To ensure early detection, specialist organizations like the National Comprehensive Cancer Network, American Cancer Society, American College of Radiology, and the American Society of Breast Surgeons recommend breast cancer risk assessment beginning as soon as 25 years old (NCCN, Spalluto et al. 2023). Within primary care, the United States Preventative Services Task Force specifically recommends that clinicians assess women with a personal or family history of breast, ovarian, tubal, or peritoneal cancer, or those with ancestry associated with BRCA1/2 gene mutations, using an appropriate brief familial risk assessment tool (USPSTF 2019). Under these guidelines, women who screen positive should be referred for genetic counseling and, if indicated, subsequent genetic testing (USPSTF 2019). Despite these evidence-based recommendations for risk-stratified screening based on validated risk assessment tools, which include but are not limited to Tyrer-Cuzick, BRCAPro, CanRisk, Breast Cancer Surveillance Consortium, and Gail Model, health system implementation and provider uptake of these recommendations have been limited (Spalluto et al. 2023, Warwick et al. 2014, Velentzis et al. 2023). This is significant because failure to systematically implement risk assessment in primary care results in higher morbidity and mortality in unidentified high-risk individuals (Orlando et al. 2013, Gramling et al. 2004, Blaes et al. 2020).

Evidence-based guidelines recommend tailored approaches for early detection based on individual risk (NCCN, Nelson et al. 2019, Nelson et al. 2019, Saslow et al. 2007, Lee et al. 2020). Women at increased risk of breast cancer benefit from enhanced breast cancer screening (earlier screening initiation, additional screening modalities) and potentially risk-reducing measures (chemoprophylaxis, prophylactic surgery) to promote earlier cancer detection and decrease breast cancer mortality (NCCN, Saslow et al. 2007, September, Amornsiripanitch et al. 2021). Supplemental MRI screening significantly increases detection of breast cancer with a sensitivity of 90%, compared with 37.5% for mammography in BRCA carriers who have a 70% risk of breast cancer (Lee et al. 2020, Armstrong and Evans 2014, Kuchenbaecker et al. 2017, Kuchenbaecker et al. 2017, Monticciolo et al. 2018, Balmana et al. 2011). Supplemental screening may additionally be recommended for individuals with hereditary cancer mutations associated with increased risk of breast cancer or for women with a lifetime breast cancer risk > 20%. In women with a 5-year risk of breast cancer > 1.67%, chemoprevention can reduce breast cancer risk by 49% (Fisher et al. 1998). Eligible women should be offered enhanced screening and risk-reduction strategies to reduce breast cancer mortality. Essential to this discussion is proactive risk assessment and subsequent communication about breast cancer risk. Primary care is ideally situated for risk assessment and informed discussion on breast cancer screening as the primary location for delivery of preventive health services.

In one study, among women identified as eligible for genetic testing, only 9.5% reported discussion on genetic testing with a clinician and 2.7% reported uptake of genetic testing (Hull et al. 2018). Primary care clinicians express support for genetic testing to identify women at increased risk of breast cancer. However, barriers in primary care to genetic testing for hereditary cancer include limited knowledge, lack of confidence in breast cancer risk assessment and eligibility criteria for genetic testing, and concern for testing cost (Conner et al. 2025, Dekanek et al. 2020, Hamilton et al. 2017). Patient perspectives within primary care on incorporating such risk assessment tools into routine care are more limited. Women with a lifetime breast cancer risk greater than 20–25% report the need for information about their individual risk and recommended screening options, yet majority are unaware of MRI screening recommendations. Despite eligibility and guideline recommendations, only approximately 20% of eligible women complete breast MRI screening, with financial barriers representing an important challenge for women referred for screening (Conley et al. 2025). Previous studies have found that the use of personalized risk scores and risk stratification are acceptable to adult women (Tan et al. 2025, Taylor et al. 2023). Acceptability of risk assessment has been as high as 85% with higher reported acceptability for enhanced screening measures (e.g. screening breast MRI) than prophylactic measures (e.g. chemoprophylaxis) (Usher-Smith et al. 2023).

The proposed study seeks to expand on existing patient perspectives on breast cancer risk assessment in primary care incorporating perspectives of both average risk women and women with an increased lifetime risk of breast cancer. Furthermore, this study seeks to go beyond identification of barriers to evaluate and understand patient preferences for the content and delivery of risk-informed communication for breast cancer to directly inform the development and implementation of breast cancer risk stratification approaches.

## Methods

Understanding patient perspectives is essential for effectively designing and implementing new clinical tools (Israel et al. 1998, Prevention ). Twenty participants were recruited, encompassing individuals at both self-reported average and high-risk for breast cancer. Qualitative data collection and analysis were conducted guided by the Informed Decision-Making Framework (IDM) (Bowen et al. 2006). This framework has been applied to cancer screening to improve patient-clinician communication, including understanding the risk of cancer, the perceived benefits and alternatives of cancer screening, values regarding the potential harms and benefits, and engaging in decision-making at a level a patient feels comfortable (Bowen et al. 2006, Champion and Jossey-Bass 2008, Rimer et al. 2004). This framework was selected based on its applicability to breast cancer risk assessment and discussion of genetic testing as well as its potential to inform future interventions to improve risk assessment in the primary care setting. The Consolidated Criteria for Reporting Qualitative Research (COREQ) checklist guided qualitative data methods and results reporting (Tong et al. 2007).

### Inclusion criteria

Inclusion criteria included women 25–75 at either average or high-risk of breast cancer with stratified recruitment to include representation of individuals across the range of breast cancer risk. Only female participants were selected based on breast cancer risk and the differential risk associated with male breast cancer. Participants self-reported their breast cancer risk at the time of the interview. Individuals were seen by the Medical University of South Carolina for their health care.

### Interviews

Participants were recruited using MyChart messages sent to women ages 25–75 with elevated breast cancer risk ICD codes, flyers available at MUSC primary care clinics in triage locations, and outreach using social media platforms. Only individuals who had not opted out to system-wide research contact were approached through MyChart outreach targeting recruitment of individuals at increased risk of breast cancer for study participation. Of the individuals contacted, 4% opted to participate (10/249). The remaining participants were identified through flyers and social media outreach which did not include risk specific messaging. Individual self-reported risk-status was not known prior to interview invitation. Twenty virtual semi-structured interviews were conducted on Microsoft Teams using audio recording. Interview duration ranged from 13 to 32 min. Interviews were conducted until saturation of depth and diversity of themes occurred. In addition to interview questions, participants provided structured feedback on a communication guide and decision aid for communication of hereditary cancer risk. Neither the interviewer nor study team had previous interactions with the participants. Study objectives were provided to interview participants prior to informed consent. Interviews were transcribed and reviewed for accuracy. Participants were not contacted following the initial interview, but were provided the opportunity to reach out about study results. Interview participants received a gift card as compensation for their time and participation. Our research team is composed of primary care clinicians, surgical oncologists, and public health researchers. Coding was completed independently and discrepancies were reviewed by consensus.

### Conceptual framework

The study was grounded in the IDM Framework (Bowen et al. 2006, Rimer et al. 2004, Damschroder et al. 2022). A structured interview guide and key constructs were developed grounded in the IDM Framework and Prochaska’s concept of decisional balance. These key constructs were previously tested for informed decision making in prostate cancer and modified for breast cancer risk and associated screening (Rimer et al. 2004, Briss et al. 2004, Shungu and Sterba 2021, Prochaska and DiClemente 1983). The interview guide was developed and revised following feedback from the research study team. Key constructs evaluated included perceived risk, perceived severity, perceived benefits, perceived barriers, and preferred forum for informed decision making (Rimer et al. 2004). Within these domains, the framework seeks to address how participants understand breast cancer, breast cancer screening, and the risk of breast cancer. Perceived risk represents a woman’s belief about her susceptibility to developing breast cancer, while perceived severity is her view on the seriousness of the disease. Similarly, perceived benefits refers to perceptions about the positive outcomes of screening, and perceived barriers are the obstacles she believes will prevent her from completing screening. The IDM framework additionally evaluates how women can participate in breast cancer screening at a personally desirable level which includes domains of decisional satisfaction, preferred formats for communication, and clinician communication preferences. During the interviews, participants also provided targeted feedback on a communication guide designed to explain genetic testing and a clinical decision aid intended for potential future use by primary care clinicians.

### Template analysis

Thematic analysis was conducted using a template analysis approach by two independent coders and discrepancies in coding were resolved through discussion to achieve consensus (Brooks et al. 2015, Crabtree 1992). Template analysis utilizes a structured template while still permitting a flexible coding process for the analysis of qualitative data. An audit trail was used with continued engagement in the data until finalization of themes (Nowell and WD 2017). Since the interview guides in this study were based on the IDM framework, this deductive/inductive approach allowed researchers to utilize an initial template developed based on the constructs of the IDM framework (i.e., deductive), while also allowing codes to arise directly from the data in the coding process (i.e., inductive) (Crabtree 1992, Ltd 2018). Coding was conducted by two independent coders (TM, JM) who both independently coded all transcripts. Following initial limited interview coding, further coding continued by consensus of themes. NVivo software (Lumivero) was used to assist in coding (Ltd 2018). A saturation of themes was reached after 20 interviews (Saunders et al. 2018).

### Ethical considerations

The study was IRB approved as exempt (Pro00140033) with a waiver of a written informed consent. All participants provided verbal consent for participation. Interview transcripts were de-identified prior to coding and analysis.

### Statistical analysis

Descriptive statistics including means and standard deviations were calculated for continuous variables and frequency data for qualitative variables.

## Results

### Demographics

The average age of participants was 44 with the majority of participants White (80%, *n* = 16) and additional participants Black or African American (20%, *n* = 4) (Table 1). All participants had college education or higher (100%). Participants were both at average risk (55%, *n* = 11) and at increased risk (45%, *n* = 9) of breast cancer based on self-report in response to the question “Have you been told by a health care team member that you are at increased risk for breast cancer?”. Three participants self-reported having a pathogenic variant that increased their risk for breast cancer.

**Table 1:** Demographic characteristics of patient population

Variable	Frequency	Percentage
Age		
25-34	4	20%
35-44	9	45%
45-54	4	20%
55-64	2	10%
65-75	1	5%
Race		
Black or African American	4	20%
White or Caucausian	16	80%
Risk		
Average Risk	11	55%
High Risk	9	45%
Education		
Eight Years or Less	0	0%
Some High School	0	0%
High School Graduate	0	0%
Some College	0	0%
College Graduate or Beyond	20	100

### Qualitative themes

Our analysis identified eight unique themes mapped to the IDM framework and three themes derived directly from the data. Interview themes were similar across average- and high-risk groups, though several distinct needs, concerns, and preferences emerged. Interview themes focused both on breast cancer risk and genetic testing. See below for description of each theme and see Table 2 for representative quotations.

**Table 2:** Exemplary quotations for themes from qualitative analysis

Theme		Average-Risk Participants	High-Risk Participants
Understands the test, the condition, personal risks, uncertainties, and information sources			
Perceived Risk	• Hereditary factors most commonly identified as increasing risk; some expressed fear of having a genetic predisposition. • Additional factors such as dietary contribution, smoking, obesity, stress, and physical were identified. • There was uncertainty about the impact of a hereditary cancer mutation.	“I think I’m aware of maybe if there’s like family history of it.. I’ve heard different things about it could be like the food you eat as well.” (INT 11) “All I know is that [the risk is] higher, but I don’t know specifically what that means.” (INT 20) “I don’t believe it means you definitely will get breast cancer. I believe it just means you are at increased risk for having it.” (INT 17)	“I know genetics can play a risk, like the BRCA gene, and I understand it is hereditary.” “You just worry about how long. Is it something that’s gonna kill you or how much longer are you gonna live with it so? Is it a death factor?” (INT 20)
Perceived Severity	• There was a lack of detailed knowledge of risk factors for breast cancer beyond family history. • Uncertainty about risk associated with mutations was common. • Many in both risk groups endorsed fear and anxiety around the possibility of a positive genetic test result and had questions about implications for self and family. • Provision of additional clinical information in some high-risk participants reduced anxiety about breast cancer risk	“I think.. if it were positive, it would negatively impact my perception of my own health, because I think I would kind of see myself as a bit of a ticking time bomb.” (INT 1) “I mean it would definitely have a big impact like mentally. So I think having the necessary resources set up like obviously having a good family support system, but also maybe get involved in some therapy.” (INT 12)	“I would be like ‘Oh my gosh, am I a ticking time bomb?’ and ‘How is it going to impact my offspring’s health?’.. Just maybe mentally it would cause some anxiety.” (INT 7) “I cried for a very long time initially, then after I’d done some research and talked to some family members and talked to some providers and I felt better about it. And honestly now like unless I have an appointment coming up, I really don’t think about it.” (INT 3) “When I met with the genetic counselor, it seemed to make me feel more positive about my cancer risk.” (INT 13)
Information Sources	• General awareness of breast cancer was high, but in-depth knowledge of genetic testing was limited. • Media, online resources, healthcare organizations, and healthcare providers were key/trusted information sources • Discussions with specialized health care providers, including genetic counselors and oncology specialists, were more often cited by high-risk participants	“I think I have always known from either my family or just kind of general knowledge ‘cause I feel like there’s a decent amount of breast cancer awareness out there.” (INT1) “I know you can look for the BRCA 1 gene and again, I believe that was through just seeing like celebrities on TV or whatnot saying that they screen positive for that.” (INT2) “Just by my own knowledge, like knowing that and then on TV commercials and all that stuff and just medical professionals to get that information from.” (INT 16)	“I was pretty well versed in all that stuff before I had seen my result, and then when I got my result, I started to, and I’m probably an outlier because I probably went too far with all this, but, I investigated my specific allele variant.” (INT6) “I guess from the people I’ve seen at {medical hospital]. And I did see a genetic counselor with my mom several years ago. And just reading online, doctor Google.” (INT 7)
Role of Population-Based Genetic Screening	• Highlighted cost differential between clinical genetic testing and consumer-based testing • Limited knowledge on the limitations of consumer-based testing	“So I know MUSC does In Our DNA and so they can detect some genetic testing… But I’ve just known that because I work here.” (INT 11)	“…when I had that 23andMe, I was just kind of like, ‘Oh, looks like I’m good,’ you know. So it was a surprise to me when I found out.” (INT 2) “The Helix test or something like my 23andMe, like those are a little bit cheaper, more affordable or free.” (INT 6)
Considers preferences			
Perceived Benefits	• Informed decision-making, preventive opportunities, peace of mind, and empowerment were identified as key benefits of genetic testing. • Negative test results were reassuring to participants. • Desire to inform relatives, especially their children, about inherited risk was a strong motivator influencing decisions around genetic testing. • Majority of participants reported that additional screening and risk-reducing measures were beneficial and reassuring.	“So that they would know maybe to begin screening earlier, to be more diligent about their breast exams or changes in their body and to just kind of overall gain some peace of mind, hopefully that you know they might not be as high of a risk as they thought.” (INT 1) “So people want to be more proactive to get those exams done. More for prevention.” (INT 10) “I think that it gives you a better idea of what your options are moving forward.” (INT 15) “Personally, I think it would be a great benefit to have that peace of mind” (INT 18)	“The risks and benefits of genetic testing was conveyed to me and I elected to undergo genetic testing because I felt that it would be the best way for me to better understand my potential risks and to take early action before disease became present and problematic.” (INT 2) “The helpful part was that the breast screening is very good. Like it’s very good at catching things early between ultrasound, mammography, and MRI.. so I felt comforted by that.” (INT 6)
Perceived Barriers	• Financial costs, limited access to care, insurance coverage, and confidentiality concerns emerged as barriers to genetic testing. • The value of free testing programs was identified as reducing barriers.	“I would say confidentiality. I feel like a lot of people don’t like doing those types of testing because they’re not sure where the information is going to be shared.” (INT 11) “Challenging, maybe because they might not want to find out ahead of time.” (INT14) “I think the cost is one thing that might deter people from getting genetic testing… I think the cost was like 400–500 dollars. Thankfully …my provider was able to, I guess, write the prior authorization to get that [waived] for me.” (INT19) “And I like to know what, instead of being a big cloud of fear. You know, I want to know the facts.” (INT 8)	“…while there are commitments being made by the medical profession, you know, to keep our information secure, data breaches happen all the time.” (INT 3) “I would be scared out of my mind. I would love to know, but I don’t want to know.” (INT 20) “Well, easy in the sense that the helix screen was free of charge because I know that those tests can cost a lot of money if you don’t have a strong family history.” (INT6) “I actually was scheduled for an appointment.but didn’t actually have the genetic testing done because part of the conversation was that they asked if I had a good life insurance policy in placed because the results of that testing could impact my ability to get life insurance in the future.” (INT 13)
Role of Family	• Concern for family members influenced decision making regarding genetic testing and subsequent screening. • Participants felt it was important to communicate risk information to family members.	“You would be concerned and scared about the future, about what that means for your family, whether you would be able to have children… If you already have children, what that means for them…” (INT 17) “If I knew that breast cancer ran in my family, I would probably be more knowledgeable to try to understand how it may affect me, my household, or my family” (INT 14)	“When I did this, my first concern was like, I have to tell my family members and I know that they can agree or disagree and not do testing or go all the way and do all the testing and the surgery. But like, I still felt like I had to tell them.” (INT 5) “And that made it helpful, because then that meant to me, that not everybody had to go get genetic testing in my family. As long as certain people came back negative…then their kids would be negative.” (INT 19) “I have a daughter of my own, so I think about her health, as well as just, you know, knowledge is power.” (INT 13)
Follow-Up Preferences	• Desire for proactive health management motivated follow-up. • Key drivers for adherence to recommended follow-up included understanding benefits, logistical convenience, uncertainty about test results, and social support. • Clear communication about time and location of scheduled follow up was desired. • Family factors were motivators to completing recommended screening and follow-up. • Individuals at high risk emphasized the importance that they placed on follow-up; however individuals at average risk more often desired additional information in particular about risks and benefits of follow-up?	“If you don’t truly understand what you’re being told, you may not follow up because you don’t understand the risk that comes with the testing results.” (INT 14) “Knowing if going to these appointments… how that shapes your risk going forward. If [it] will diminish the risk, by how much?” (INT 17) “If I tested positive, I would 100% do whatever screenings.” (INT 1)	“I just had a new little daughter and I wanna see her graduate college. So doing anything I can to make that happen.” (INT 9) “There’s just not enough information on my variant to say whether or not I should have surgery sooner rather than later…”(INT 9) “I don’t skip them, I want to stay on top of things.” (INT5) “I’ve elected to continue with screening because of my family’s history… just about everybody that’s had it that’s been a male, has had prostate cancer. I did have one family member that ended up getting metastatic cancer,” (INT 6)
Participates in decision at a personally desirable level			
Preferred Forum/Formats for IDM	• Directed communication with the health care team was valued. • Participants highlighted the need to have sufficient time for discussion and questions. • Delivery of information prior to an appointment was suggested to improve discussion. • Direct conversations, physical handouts, and online resources were preferred formats.	“I think for me, just like speaking directly with my physician…and then maybe giving me a pamphlet that has more information.” (INT 12) “Probably a discussion at a check-up with my primary care physician and then a handout or video I could watch in MyChart.” (INT 17)	“I feel that one of the things that is starting to slip away from medicine in general is that interest in communication and human connection. I think that, that ensuring that the provider has enough time to speak with each patient and answer their questions is really important and I think that for certain patients, having information backed up with like a written form, like a pamphlet or like an e-mail or whatever is helpful…” (INT 3) “Meeting the patient at their level of education and comfort and just kind of explain based on their medical history and chart why the provider thinks genetic testing would be appropriate.” (INT 9) “Potentially if you are going in for a routine or yearly kind of thing maybe you’d be…presented with some materials either by mail or in Mychart that you might want to look at before you get to your provider, and so that you would have an idea to discuss it at your visit.” (INT 2)
Clinician Communication	• Limited information is provided by primary care physicians about risk and genetic testing. • Participants desired clinician-initiated conversations about genetic risk assessment to be more routinely integrated into primary care. • There was a need identified for more comprehensive, clear, empathetic, personalized conversations with clinicians regarding testing rationale, procedures, costs, results, follow-up plans, impact on insurance, and available resources. • Information about next steps and recommended follow-up was valued. • In addition to the facts and information, physician recommendation was valued as part of the informed decision- making process.	“I haven’t received any [information about risk and testing] at all [from my primary care clinician].” (INT 11) “…if the conversation was started for me, then I would start asking questions and I would be more willing to intake information.“ (INT 18) “…a little bit of warmth and like maybe some concern coming from the provider or compassion would be important…” (INT 18) “Not only the nuts and bolts of how, where, and what the results mean, but… stories about women who have had the testing and what happened to them and what did they do and what did it mean for their lives.” (INT 8) “Presented with the information ‘Ok you tested positive for this screening and now this is what we are going to do‘ rather than ’You tested positive, so now you kind of you know you’re at higher risk‘. I would want the follow up plan outlined for me because otherwise, I think I would just panic.” (INT 1)	“I don’t really think I’ve ever received [any information] from my primary care.. I guess I just wasn’t really aware that [risk assessment] can be done just as a part of routine maintenance.” (INT 2) “I was told about kind of the risks and benefits and what information it can provide and not provide… The logic and the reasons for doing the genetic testing were very, very apparent and clear.” (INT 3) “I would say that that interaction [with genetic counseling] was frankly very positive, very informative. I felt very heard and very seen by the team that was doing that.” (INT 3) “I would have liked to have known more information about my variant, like in population studies.” (INT 6)
Decisional Satisfaction	• Participants desired information in a format that was consistent with their values and helped them make an informed decision. • High-risk participants endorsed decisional satisfaction with receiving genetic testing for hereditary cancer	“Yes I do….I just want to be aware. It’s already a scary time and just knowing what are the possibilities of something, of what other cancers I may have in my body or be more prone to is something that I want to know for my own knowledge” (INT19)	“I had to have all of these tests done and the ovaries out and the [endoscopy]…but to me I’d rather go through those things and lower my risk” (INT 2) “The decision for me wasn’t so hard, but when I tried to tell other people to be aware, it was” (INT 4)

### Perceived risk

Perceived risk focused on a participant’s subjective evaluation of her risk of developing breast cancer (Shungu and Sterba 2021). Hereditary factors, particularly a family history of breast cancer, were consistently recognized as risk factors across both groups. Environmental and lifestyle factors, such as smoking, obesity, diet, stress, and alcohol consumption were also identified, though average-risk participants often had more limited detailed knowledge beyond family history compared to high-risk participants. Across groups, participants commonly described a lack of clear understanding of the specific increased risk associated with certain genetic mutations.

### Perceived severity

Perceived severity is a woman’s feelings on the seriousness of contracting breast cancer and potential complications. Participant perspectives were similar in perceived severity between average-risk and high-risk participants. This included the fear and anxiety associated with a positive genetic test result, with some comparing a high-risk status to a “ticking time bomb”. Additional concerns were expressed about the negative impact of risk on perception of health. Participants in both groups also expressed uncertainty about severity and desired more concrete information about the actual impact of increased risk. Impact on family, particularly children, was a significant concern, potentially influencing future reproductive decisions. If a genetic variation was found, participants highlighted the need for support from both family and behavioral health resources.

High-risk participants offered nuanced insights into perceived severity. While fear and anxiety were present, some reported that positive genetic test results didn’t significantly alter their daily lives or overall perception of health, and that initial emotional distress lessened over time. The impact of family members’ experiences with cancer, especially deaths, influenced their perceptions of severity.

### Information sources

Information sources focused on participant’s sources of information for both breast cancer screening and genetic testing. For average-risk individuals, general awareness about breast cancer stemmed from family, friends, public health campaigns, and media (television, news articles, celebrity stories). They generally had limited in-depth knowledge about hereditary breast cancer genetic testing, though a basic familiarity with its existence and genes like BRCA1 was present. Individuals working in healthcare or research environments reported more specific knowledge.

High-risk participants reported more active information-seeking behaviors citing in particular online resources such as the National Institute of Health and the Susan G. Komen foundation website. Healthcare providers and genetic counselors were common information sources, though the depth of discussion varied. Family experiences, particularly having a mother or other close relatives with breast cancer, strongly increased awareness and proactive information seeking. Similar to average-risk participants, media also played a role. High-risk participants demonstrated a wide range in their depth of understanding about genetic testing, often deeper due to direct experience.

### Role of direct-to-consumer and population-based genetic testing

Direct-to-consumer genetic testing is a patient-initiated process, with individuals obtaining testing directly from commercial companies. Results are not automatically shared with healthcare clinicians. Testing approaches can vary widely, from targeted variant analysis to more comprehensive genomic testing. Because some direct-to-consumer tests are not all clinically validated, confirmatory clinical testing may be required. Population-based genetic screening is healthcare system initiated and offered to all individuals regardless of personal or family history. These tests are typically clinical grade and a structured pathway for clinical follow-up is available when indicated. The institution where this study was conducted had a population-based genetic screening program at the time of participant interviews. Participants in this study identified the impact population-based screening programs as well as direct-to-commercial screening programs. Participants frequently reported their first encounter with genetic testing through direct-to-consumer companies, often driven by curiosity about ancestry, or as part of research studies. High-risk participants raised concerns about the limitations of commercial testing, particularly its limited scope and sensitivity for breast cancer genes, especially for non-Ashkenazi Jewish ancestry, which could provide a false sense of security. Some reported receiving negative results from direct-to-consumer testing, only to later receive positive results for cancer-related genes through more comprehensive clinical grade testing. The affordability or even free nature of population-based tests was seen as a significant advantage. Participants desired further education on the limitations of commercially available testing and on the cost and availability of genetic testing.

### Perceived benefits

Perceived benefits refers to participant’s perceptions of the positive outcomes derived from completing genetic testing. Both average- and high-risk participants identified informed decision-making about future screening and preventative actions as a key benefit of genetic testing. The potential for prevention through surgery or medication to decrease the likelihood of developing cancer, and improved outcomes through early detection, were highly valued. Peace of mind, whether from a negative genetic testing result or from simply knowing their genetic risk, was also identified as a benefit. High-risk participants emphasized empowerment as a benefit, viewing knowledge of genetic risks as empowering a more proactive approach to their health. They also focused on their ability to help other family members understand their own potential risks.

### Perceived barriers

Participants provided feedback on the obstacles to completing genetic testing. Participants in both the average-risk and high-risk groups endorsed similar perceived barriers to genetic testing with a primary focus on confidentiality of health information, fear of a positive test result, and concern for cost of testing. Confidentiality concerns included worries about data storage, access, sharing with third parties (government, insurance), data breaches, and profiling. Fear of results was a significant psychological barrier, with some preferring not to know to avoid potential anxiety. Financial barriers were a pervasive concern across both risk groups, including the cost of testing, uncertainty about insurance coverage, and the availability of free or low-cost programs. Access issues, such as transportation difficulties and the need to take time off work, were also noted, with recommendations for on-site testing at primary care or OB/GYN offices or mobile testing. Insurance discrimination concerns regarding future life, health, and disability insurance were particularly noted. High-risk participants highlighted logistical difficulties related to navigating insurance coverage and scheduling appointments, likely due to direct experience with these processes.

### Role of family

The role of family theme addresses family considerations and their impact on decision making regarding genetic testing. For both average- and high-risk participants, concern for children, especially daughters, and the impact of breast cancer risk on family planning were significant themes. A close family history of breast cancer was a strong motivator for interest in genetic testing and becoming more informed. High-risk participants additionally uniquely emphasized the responsibility to inform family about a positive genetic test result, acknowledging that family members have the autonomy to decide whether or not to pursue testing and preventative measures.

### Follow-up preferences

Participants provided information on the factors that would influence their decision to engage in follow up testing and to attend follow up appointments if a high-risk status or a hereditary cancer mutation was identified. For both average- and high-risk individuals, receiving concerning test results was identified as a strong motivator for scheduling and attending follow-up appointments. Participants emphasized that having comprehensive information about results and their implications was vital for making informed decisions about next steps. Practical considerations, such as convenient appointment times and locations, were noted as important facilitators. Across both groups, a general desire for proactive health management also served as a positive motivator.

Among average-risk individuals, understanding the benefits of follow-up, such as how it could reduce risk and lead to better treatment and survival outcomes, was a key motivator. They also valued social and emotional support, reporting that having a supportive companion during appointments was beneficial for emotional well-being, information absorption, and potentially increasing follow-up adherence.

High-risk participants raised several unique concerns. Lack of variant-specific information and uncertainty about the implications of rare genetic variants often complicated decisions regarding follow-up. Body image considerations related to preventative mastectomy were noted. While many high-risk individuals described relying on their clinicians to assist with follow-up recommendations, the decision to pursue follow-up testing and preventative measures was widely recognized as a personal choice. These decisions were often deeply influenced by individual circumstances and values, including future goals such as witnessing a child’s graduation.

### Preferred forum/formats for informed decision making

Participants identified their preferred formats to receive information about genetic testing. Direct, personalized communication especially with their physician was preferred by both average- and high-risk participants. Physical handouts (pamphlets, flyers, brochures) and online resources, particularly links sent through MyChart, were desired to reinforce information.

High-risk participants expressed a preference for pre-visit resources sent via mail or MyChart, allowing them to review materials and prepare questions for a more informed discussion during their appointment. They also emphasized the importance of sufficient time for questions during appointments.

### Clinician communication

The clinician communication theme identified previous information receipt and informational needs both for risk communication as well as discussion on genetic testing. Across both average- and high-risk groups, participants emphasized the importance of clear communication and empathetic delivery of information from clinicians. A consistent theme was the desire for rationale - understanding why a particular screening or genetic testing recommendation was made. Participants wanted detailed information about the procedures involved, including what tests entail (e.g., blood drawn, specific procedures), associated costs, turnaround time for results, interpretation of results, and testing accuracy. The provision of follow-up plans and next steps, both immediate and long-term, was highly desired, particularly at the time of receiving positive genetic testing results, to alleviate anxiety and provide a sense of direction. They sought contextual information, such as the likelihood of developing cancer, survival rates, and outcomes of others in similar situations. Access to support and resources, including counseling and support groups, with proactive facilitation of connections, was greatly appreciated. Reassurance about confidentiality of genetic testing results was also consistently valued.

Participants desired clinician-initiated conversations about breast cancer risk and screening, with only one of the participants across both risk levels reporting they had discussed these topics with their primary care clinician. This highlights a critical opportunity for proactive engagement. Participants also emphasized using plain language to ensure results were easily understood.

High-risk participants offered additional insights. Some expressed surprise that genetic risk assessment is not a more routine part of preventative care. They sought more information about their specific genetic variants and guidance on lifestyle adjustments based on their increased risk. Positive experiences with genetic counseling were reported, with interactions described as thorough, unrushed, informative, and supportive, often enhanced by visual aids like family trees. The desire for ongoing support and guidance after the initial genetic counseling session was strong, particularly for navigating decisions about preventative surgeries and managing their aftermath.

### Decisional satisfaction/values consistency

Following review of a clinical decision aid on genetic testing, participants were asked about their capacity for decision making about genetic testing and its alignment with their values and preferences. If an individual had previously discussed or completed genetic testing, she had an opportunity to comment on decisional satisfaction with this encounter. Both average- and high-risk participants generally reported favorable impressions of a clinical decision aid and that the information provided in an aid would assist them in making an informed decision about genetic testing. For the majority, the decision to receive or not receive genetic testing for hereditary cancer should be consistent with their personal values and preferences. However, one individual reported financial constraints influenced her decision, stopping her from pursuing genetic testing that was otherwise aligned with her values and preferences.

## Discussion

### Principle findings

Implications of IDM in delivery of tailored screening services will become increasingly important with expanding evidence for risk-tailored cancer screening, polygenic risk scores, and population genomics (Esserman et al. 2025, Pashayan et al. 2020, Roberts et al. 2023, Padrik et al. 2025). These strategies have been employed not only in breast cancer screening but also other screening modalities including prostate cancer, colorectal cancer, and lung cancer (Kastrinos et al. 2023, Marcus et al. 2016, Callender et al. 2019, Huntley et al. 2023). The current study evaluated patient preferences around communication of breast cancer risk and genetic testing in both individuals at average and increased risk of breast cancer.

Primary care practices provide the majority of screening services including cancer screening with opportunity for improved delivery of breast cancer risk assessment, risk communication, and early identification of individuals eligible for genetic testing for hereditary cancer mutations. Despite this potential opportunity, in this study, only one of the participants had previously discussed breast cancer risk with their primary care clinician. Many women at increased risk for breast cancer identified desiring a multidisciplinary care team including gynecologists, geneticists, and oncology specialists. This presents an opportunity for improved risk assessment and communication at the primary care level as part of systematic and comprehensive discussions on breast cancer screening recommendations and improvement in coordination of care.

In this study, participants valued direct, clear communication with their physicians with provision of specific information on breast cancer risk, subsequent screening recommendations, and potential impact on family members. Although some knowledge was present about hereditary breast cancer risk, future interventions designed to improve risk communication will need to have an educational component to assist participants with increasing their knowledge on breast cancer risk and some of the factors that might impact it. This need for education persisted even in the high-risk group when there had been previous discussion of breast cancer risk including with genetic counselors and hereditary cancer specialists. Finally, participants in both groups identified the potential fear and anxiety associated with delivery of information about an increased risk of breast cancer could be alleviated in part by specific risk communication tailored. These findings have direct potential impact on primary care changes around risk communication and genetic testing in primary care as well as in the design of future interventions to support this process. Based on the favorable responses to the clinical decision aid, future studies plan to evaluate its potential implementation into primary care.

### Comparison to prior work

Although previous research has been conducted about risk and genetic testing in individuals with cancer, limited research has been conducted on patient perspectives on knowledge, risk perception, and communication about genetic testing in individuals at increased risk for breast cancer in the primary care settings. Primary care is ideally situated for risk assessment and informed discussion on breast cancer screening; however, majority of participants in this study did not report discussion of breast cancer risk with their primary care clinicians. Primary care clinicians report the value of screening for inherited cancer risk; however, they also report low use of and confidence in the ability to use validated risk assessment tools (Gramling et al. 2004, Amornsiripanitch et al. 2021, Corbelli et al. 2014, Bellhouse et al. 2021). Only 40% of PCPs report having ever used a risk assessment tool and only 8.6% reported confidence in their ability to use these tools to identify women at increased risk of breast cancer (Corbelli et al. 2014, Bellhouse et al. 2021). Previously identified barriers include inadequate time, insufficient training, lack of clinician knowledge, competing health priorities, patient preferences and lack of tools and resources (Spalluto et al. 2023, Bellhouse et al. 2021). Although primary care delivery of risk assessment has been limited, it has previously been shown to be acceptable to the general population (Usher-Smith et al. 2023, Kaplan et al. 2021, Kaplan et al. 2014).

Themes regarding communication about breast cancer for individuals without a cancer history are similar to those found in previous studies in individuals with breast and colon cancer. With the increasing role of genetic testing and precision medicine both for risk assessment and cancer diagnosis follow-up, it is important to understand barriers, facilitators, and information needs for risk assessment. Consistent with previous research, the present study identified a high level of public interest in genetic testing, contrasted by a self-reported lack of knowledge among these same participants (Peterson et al. 2018, Peterson et al. 2016). Therefore, communication strategies about breast cancer risk and genetic testing should address informational needs.

McCormick et al. evaluated decisional satisfaction regarding a population-based genetic testing study. Participants in this study reported low levels of negative response and decisional regret after testing (McCormick et al. 2022). Some subgroups of participants were reported to have increased decisional regret, including individuals with lower educational attainment and other disadvantaged backgrounds (McCormick et al. 2022). Similarly, decisional regret for genetic testing was not reported by participants in this study who had opted to participate. Therefore, it is critical that future studies evaluate a diverse group of individuals to best understand health communication strategies across the population for genetic risk (Kaphingst et al. 2019).

Themes identified in this study included perceived barriers on the system and individual levels. System level barriers commonly reported in other studies include cost of testing and insurance considerations including life insurance (Peterson et al. 2018, Tiernan et al. 2022, Campbell-Salome et al. 2021). Individual-level barriers identified in the current study aligned with previous research and included anxiety and fear about potential results, as well as follow-up steps. Although anxiety and fear have been identified as barriers to considering genetic testing, previous literature has identified that cancer worry and higher perceived risk were associated with increased interest in completing hereditary cancer genetic testing (Peterson et al. 2018, Cilli et al. 2025). Therefore, communication and resources that accurately present risk, while mitigating fear through information and follow-up strategies, must be considered in health communication strategies around genetic testing. Even when genetic testing has been completed, psychological support from clinicians, genetic counselors, and behavioral health team members is needed when information about a hereditary cancer mutation is delivered. This need was identified in both average-risk participants and high-risk participants in the current study. Given that limited clinician knowledge can increase patient anxiety and uncertainty, additional clinician training and improved knowledge will best support this process (Tiernan et al. 2022, Campbell-Salome et al. 2021, Cilli et al. 2025, Ankersmid et al. 2024).

Both this study and previous research have identified the important influence of family in impacting decision making regarding genetic testing for hereditary cancer risk (Kuo et al. 2023). In this study, participants focused more on the potential impact on offspring, either in family planning or future risks and needs of their children. This finding aligns with previous work by Kaphingst et al. in a study of young women following treatment for breast cancer (Kaphingst et al. 2019, Kaphingst et al. 2018). These women were similarly very interested in receiving genetic test results that would impact their preventive care and test results that might affect the health of their children (Kaphingst et al. 2018).

The current study adds to existing literature by reinforcing barriers, facilitators, and communication preferences in individuals identified as being at average and increased risk. The study provides a range of perspectives on the psychological impact of genetic testing, both for individuals considering testing and those who have already received results. This includes the potential consequences of worry and anxiety, and additional concerns about finances and confidentiality. Participants value discussions with their primary care clinicians about risk assessment. Participants also desired varied educational formats and highlighted the need for adequate time to have these conversations. Future interventions should focus on improving patient communication while addressing clinician-reported barriers, particularly time constraints (Jong et al. 2025). Strategies to improve delivery of risk information and to promote discussion of genetic testing can be informed by both the findings of this study and by previous research on other populations, including Lynch syndrome and those with a cancer diagnosis. These studies demonstrate the essential role of integrating communication strategies to enhance shared decision-making in discussions about cancer risk and genetic testing (Kaphingst et al. 2019, Ankersmid et al. 2024).

As the scope and accessibility of genetic testing for hereditary cancer including breast cancer continues to expand, primary care physicians are uniquely positioned to identify patients who may benefit from these advances or to assist in navigation of subsequent test results. Roles of a primary physician may encompass the completion of risk assessment for hereditary cancer, referral to genetic counseling, participation in population based genetic testing programs, and continuation of surveillance for patients with known pathogenic mutations (NCCN , Spalluto et al. 2023, Force et al. 2019, Hull et al. 2020). Individuals additionally often present to their primary care clinician for interpretation and follow up recommendations on not only for clinical genetic test results but also for direct-to-consumer testing (Knapp KBaE 2018). Primary care clinicians should be prepared to navigate guideline-based risk assessment for hereditary breast cancer and facilitate referrals to genetic counselors (Tan et al. 2025, Taylor et al. 2023, Usher-Smith et al. 2023). Educational initiatives may be needed to address the previously identified existing training gaps. This study focuses on patient perspectives to optimize primary care delivery of systematic breast cancer risk assessment and support implementation of guideline-based care for hereditary cancer testing. By integrating patient experiences with the existing literature demonstrating gaps in primary care clinician education, the findings may inform both targeted educational interventions and health system strategies to support the implementation these processes.

### Strengths and limitations

This study provides insights from a cohort of participants from one health system with a variety of backgrounds including those at average risk and increased risk of breast cancer. Participants self-reported their breast cancer risk status. Therefore, findings may reflect perceived rather than objectively measured risk. However, transcript review supported high-risk classification based on recruitment using high-risk ICD codes, reported hereditary cancer mutations, and participation in enhanced screening protocols (annual MRI, early screening initiation, or follow-up in a high-risk breast clinic). For participants who had previously completed genetic counseling, there is the potential for recall bias, in particular in review of the decision aid. Additional work would benefit from a larger study to confirm the generalizability of these findings both in terms of broader recruitment and a larger geographic area. Finally, as the sample was a well-educated population, future work should be inclusive of individuals with more diverse educational backgrounds.

## Conclusion

Patient-centered communication and accessible, comprehensive information are key for successfully integrating breast cancer risk assessment into routine primary care practice. Our findings underscore the critical need for clinicians to initiate breast cancer risk conversations, communicate clearly and empathetically, and offer detailed information and follow-up about the rationale, process, and implications of breast cancer risk assessment and genetic testing.

## Data Availability

The data that support the findings of this study are not openly available due to reasons of sensitivity and are available from the corresponding author upon reasonable request. Data are located in controlled access data storage at the Medical University of South Carolina.
